# Identification of hub genes and key pathways of paraquat-induced human embryonic pulmonary fibrosis by bioinformatics analysis and *in vitro* studies

**DOI:** 10.18632/aging.203570

**Published:** 2021-09-27

**Authors:** Xiangxia Zeng, Jinlun Hu, Mei Yan, Chunming Xie, Weigan Xu, Qiaohua Hu, Jinxia Feng, Zi Cong Gu, Yue Fu

**Affiliations:** 1 Department of Respiration, First Affiliated Hospital of Guangzhou Medical University, Guangzhou 510120, Guangdong, China; 2 Department of General Medicine, The First People’s Hospital of Foshan, The Affiliated Foshan Hospital of Sun Yat-Sen University, Foshan 528000, Guangdong, China; 3 The Poison Treatment Centre of Foshan, Foshan 528000, Guangdong, China; 4 Department of Emergency Medicine, The First People’s Hospital of Foshan, The Affiliated Foshan Hospital of Sun Yat-Sen University, Foshan 528000, Guangdong, China

**Keywords:** paraquat, pulmonary fibrosis, bioinformatics analysis, *in vitro* experiments

## Abstract

Objective: Paraquat (N,N0-dimethyl-4,40-bipyridinium dichloride;PQ) is a highly toxic pesticide, which usually leads to acute lung injury and subsequent development of pulmonary fibrosis. The exact mechanism underlying PQ-induced lung fibrosis remain largely unclear and as yet, no specific treatment drugs have been approved. Our study aimed to identify its potential mechanisms of PQ-induced fibrosis through a modeling study *in vitro* studies and bioinformatics analysis.

Methods: Gene expression datasets associated with PQ-induced lung fibrosis were obtained from the Gene Expression Omnibus, wherefrom differentially expressed genes (DEGs) were identified using GEO2R. Functional enrichment analyses were performed using the Database for Annotation Visualization and Integrated Discovery. The DEGs analyzed by a protein–protein interaction network was constructed with the Search Tool for the Retrieval of Interacting Genes database. MCODE, a Cytoscape plugin, was subsequently used to identify the most significant modules. The expression of the key genes in PQ-induced pulmonary fibrotic tissues was verified by reverse transcription-quantitative PCR (RT-qPCR).

Results: Two datasets were analyzed and revealed 92 overlapping DEGs. Functional analysis demonstrated that these 92 DEGs were enriched in the ‘TNF signaling pathway’, ‘CXCR chemokine receptor binding’, and ‘core promoter binding’. Moreover, nine hub genes were identified from the protein–protein interaction network formed from the DEGs. These results suggested that the TNF signaling pathway and nine hub genes are possibly involved in PQ-induced lung fibrosis progression.

Conclusions: This integrative analysis identified candidate genes and pathways potentially involved in PQ-induced lung fibrosis, and could benefit future development of novel approaches for controlling and treating this disease.

## INTRODUCTION

Paraquat (PQ) has become a widely used herbicide in agriculture for its ability to rapidly kill leaf weeds with non-selective characteristics [1, 2]. As a harmful substance to humans and livestock, PQ poisoning has become a common cause of deaths by pesticide poisoning and has a fatality rate of 38.08% [2]. PQ intoxication leads to health damage in human health and causes multiple serious diseases, such as severe pulmonary inflammation, edema, and pulmonary fibrosis, which have high mortality rate and lack effective therapeutic strategies [3, 4]. Moreover, the extremely high toxicity and lethality of PQ impedes effective research, and the identification of the mechanisms underlying PQ-induced pulmonary fibrosis remains challenging. Thus, the accurate determination and control of the highest non-lethal concentration of PQ is a crucial prerequisite for further research on PQ-induced pulmonary fibrosis.

Microarray technology helps determine mRNA profiles and provides a comprehensive and systematic analysis of disease processes, including those involved in pulmonary fibrosis [5]. In addition, some studies have revealed that PQ intoxication may cause alterations at the genome level [5–8]. Thus, integrative analyses of genes and pathways associated with lung fibrosis may provide insights into potential therapeutic targets and diagnostic biomarkers for PQ-induced pulmonary fibrosis.

This study identified the highest non-lethal concentration of PQ usable to induce pulmonary fibrosis *in vitro* studies through testing collage I protein and α-SMA expression (α-Smooth Muscle Actin), paving the way for further studies. Moreover, we analyzed differentially expressed genes (DEGs) during PQ intoxication, and Hub genes were determined from a protein–protein interaction (PPI) network to help guide future. This integrative method is the first to identify candidate genes and pathways involved in PQ intoxication and the highest non-lethal concentration of PQ usable to induce pulmonary fibrosis *in vitro* studies.

## MATERIALS AND METHODS

### Establishment of *in vitro* studies

The human embryonic pulmonary fibrosis cell line (MRC-5) was purchased from the cell bank of the Institute for Occupational Diseases in Guangdong Province and cultured in DMEM supplemented with 10% fetal bovine serum (Invitrogen, USA), 25 μg/mL penicillin, and 25 μg/mL streptomycin (Invitrogen, USA). The cells were cultured at a constant temperature of 37° C in an incubator under 5% CO2. MRC-5 cells in logarithmic growth phase were digested to prepare a single-cell suspension and were inoculated at a density of 5×103 cells/well into cultures performed in 96-well plates and grown at 37° C overnight. PQ at concentrations of 0 (negative control), 50, 100, 150, and 200 μmol/L were used to treat cells for 24, 48, and 72 h. Fifty microliters of MTT (5 g/L) solution were added to the wells, and the cells were further incubated at 37° C for four hours. After incubation, 150 μL of a DMSO solution was added to each well. Subsequently, a volume of CCK-8 reagent equivalent to 72% of the medium volume was added to the 72-h culture, and was incubated for one hour. Then, the absorbance at 450 nm was measured with a microplate reader, and the values were used to construct the growth curves. This experimental protocol did not require any ethical approval because it was performed *in vitro* studies.

### Western blot analysis

Western blot analysis was applied to quantify collagen I, collagen III, and SAM proteins in lung tissues. Total proteins (Sigma, USA) were extracted from the cell cultures according to the manufacturer's instruction. The proteins separated by SDS-PAGE were transferred onto a PVDF membrane (300 mA, 80 min). After transfer, the target band was sealed with 5% TBS. The PVDF membrane was placed into a small ziplock bag containing 3 mL of a solution with horseradish peroxidase-labeled secondary antibody at room temperature for 50 min under gentle agitation on a shaker. The PVDF membrane was washed three times for 10 min with TBST. The solutions A and B from the ECL kit were mixed in equal volumes in 1.5 mL microtubes in the dark. After one minute, the PVDF membrane previously incubated with the above antibodies was placed facing up onto a plastic wrap, and the mixed liquid was poured onto the membrane. After one minute, the PVDF membrane with plastic wrap was placed into a medical X-ray film cassette. The X-ray film was placed onto the film, and the X-ray film cassette was closed. The time of reaction was monitored and the time of exposure, usually ranging from few seconds to few minutes, was adjusted according to the strength of the signal. On some occasions, a table showing the time gradient could be used to select the best exposure time. Next, the X-ray film cassette was opened, and the film was taken out and quickly immersed into the developing solution. Once the bands had appeared and were obvious, the film was washed in water and then transfer to the fixing solution to terminate the development process.

### RT-PCR

Total cellular RNA was extracted with a kit, according to the manufacturer’s instructions (Thermo, Shenzhen, China). The sequences of the primers used to detect the internal control SMA cDNA were as follows: forward, CCTTGAGAAGAGTTACGAGTTG; reverse, TGCTGTTGTAGGTGGTTTCA; length of the amplification product, 122 bp. The sequence of the primers used to detect the 18 srRNA were forward, CCTGGATACCGCAGCTAGGA; reverse, GCGGCGCAATACGAATGCCCC; length of the amplification product, 140 bp. The synthesized primers were purchased from Shenzhen Thermo Co., Ltd. (Shenzhen, China). The relative mRNA expression was calculated using the 2–ΔΔCq method and normalized to the internal reference gene GAPDH.

### Microarray data

The data were screened and analyzed by two contributors according to the following criteria: i) The sample was from Homo sapiens; and ii) a comparison showing high levels of alpha-SMA in lung fibrosis compared to healthy donors (negative control) was conducted. Datasets GSE40839 and GSE53845 were retrieved from the GEO (http://www.ncbi.nlm.nih.gov/geo; version 2.0) database for analysis [9, 10]. In the GSE40839 and GSE53845 datasets, the human samples were enriched with study.

### Identification of DEGs

DEGs were analyzed using GEO2R (http://www.ncbi.nlm.nih.gov/geo/geo2r; version 2.19.4) [11], according to the methods published by Wang et al. in 2019 [12]. Probe sets without corresponding gene symbols or genes with >1 probe set were averaged. Samples with an absolute value of log fold-change >1 and a p-value of P < 0.05 were retained as DEGs.

### Functional enrichment analysis

We assessed the biological characteristics and functional enrichment of the candidate DEGs according to the methods described by Wang et al. [12]. The functional enrichment analysis was performed using Database for Annotation (https://david.ncifcrf.gov/; version 6.8). Results with P < 0.05 were considered significant. Additionally, Circos, a visualization software (version 0.1.1) was used to present the data [13], and a Venn diagram was plotted using a dedicated online tool (http://bioinformatics.psb.ugent.be/webtools/Venn/; version 1.0).

### PPI network construction and module analysis

A PPI network including the DEGs was constructed using the Search Tool for Retrieval of Interacting Genes (STRING) database (https://string-db.org/cgi/; version 11.0). Following the methods from Wang et al. [12], interactions with a combined score >0.4 were considered significant. The results were visualized using Cytoscape software (version 3.7.1) [14]. MCODE, a Cytoscape plugin, was used to identify the most significant modules. The selection criteria were as follows: MCODE score ≥ 3, degree cutoff = 2, node score cutoff = 0.2, and max depth = 100.

### Statistical analysis

All statistical analyses of the results were performed using SPSS 20.0 software. All data are presented as the mean ± SD. P < 0.05 was considered to indicate a statistically significant difference.

### Data availability statement

The data used to support the findings of this study are available from the corresponding author upon request.

## RESULTS

### *In vitro* model of MRC-5 cells induced by PQ

**Figure 1:**
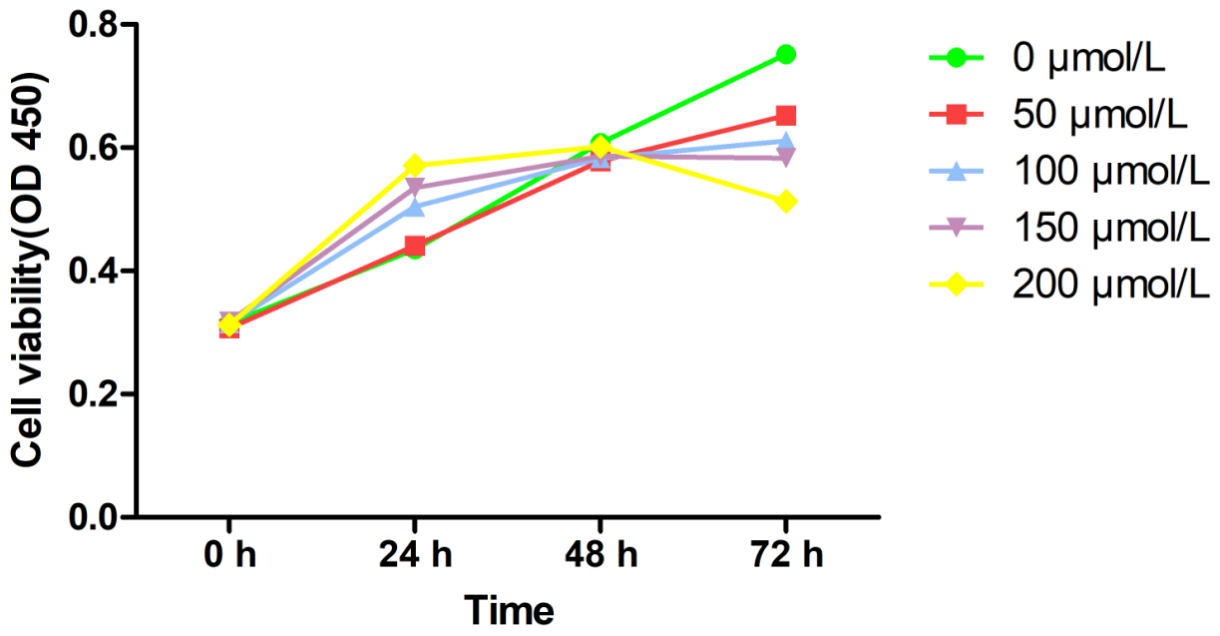
Cell viability of PQ at different concentrations on MRC-5 cells by CCk-8 assay.

The CCK-8 method was used to assess MRC-5 cells’ viability at different concentrations on PQ, to determine PQ toxicity (Figure 1). After 48 hours of treatment, upon increase of PQ concentration, MRC-5 cells showed significant proliferation. With 200 μmol/L of PQ, the MRC-5 cells proliferated significantly. However, beyond 48 h, the proliferation of the MRC-5 cells was strongly inhibited. It could be seen that the highest non-lethal concentration of PQ was 200 μmol/L, and the time window before cytotoxicity was 0–48 h.

### Western blot analysis

**Figure 2:**
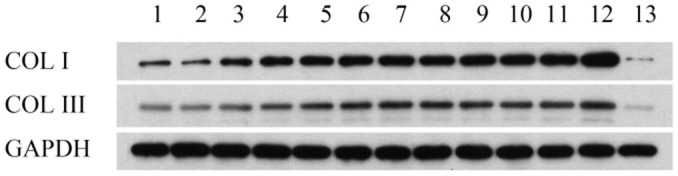
**Collage I and collage III protein expression in lung tissues detected by western blot analysis.** Lane 1, control group; lane 2, PQ with concentrations of 50 μmol/L was selected for 24h; lane 3, PQ with concentrations of 50 μmol/L was selected for 48h; lane 4, PQ with concentrations of 50 μmol/L was selected for 72h; lane 5, PQ with concentrations of 100 μmol/L was selected for 24h; lane 6, PQ with concentrations of 100 μmol/L was selected for 48h; lane 7, PQ with concentrations of 100 μmol/L was selected for 72h; lane 8, PQ with concentrations of 150 μmol/L was selected for 24h; lane 9, PQ with concentrations of 150 μmol/L was selected for 48h; lane 10, PQ with concentrations of 150 μmol/L was selected for 72h; lane 11, PQ with concentrations of 200 μmol/L was selected for 24h; lane 12, PQ with concentrations of 200 μmol/L was selected for 48h; lane 12, PQ with concentrations of 200 μmol/L was selected for 72h.

**Figure 3:**
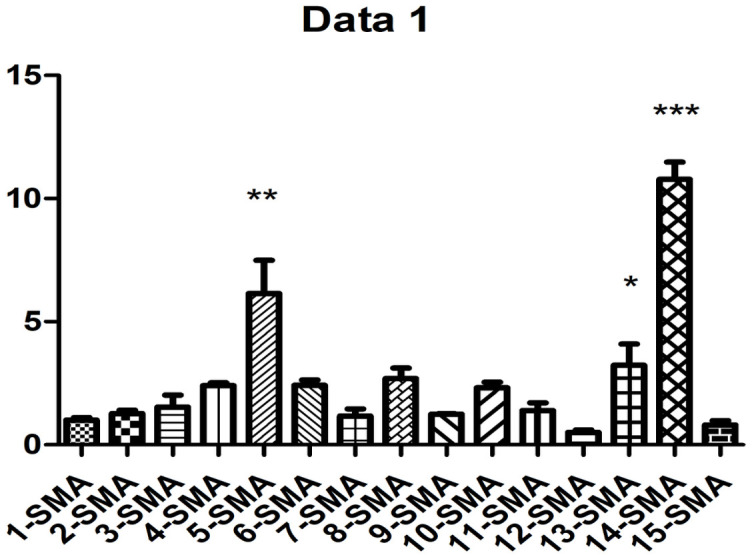
**PQ-induced α-SMA expression.** 1, control group was for 24h; 2, control group was for 48h; 3, control group was for 72h; 4, PQ with concentrations of 50 μmol/L was selected for 24h; 5, PQ with concentrations of 50μmol/L was selected for 48h; 6, PQ with concentrations of 50μmol/L was selected for 72h; 7, PQ with concentrations of 100μmol/L was selected for 24h; 8, PQ with concentrations of 100 μmol/L was selected for 48h; 9, PQ with concentrations of 100 μmol/L was selected for 72h; 10, PQ with concentrations of 150 μmol/L was selected for 24h; 11, PQ with concentrations of 150μmol/L was selected for 48h; 12, PQ with concentrations of 150 μmol/L was selected for 72h; 13, PQ with concentrations of 200 μmol/L was selected for 24h; 14, PQ with concentrations of 200 μmol/L was selected for 48h; 15, PQ with concentrations of 200 μmol/L was selected for 72h.

As shown in Figure 2 and Table 1, western blot analysis indicated that the collagen I protein was expressed in lung tissues treated with PQ at 200 μmol/L for 48 hours. In addition, as shown in Figure 3, the analysis by western blot demonstrated that PQ treatment induced a pronounced increase in the levels of α-SMA protein, with a peak of expression at 200 μmol/L of PQ lasting for 48 h. These results are indicative of a differentiation of the lung fibroblasts into myofibroblasts. This differentiation process has been associated with the development of lung fibrosis.

**Table 1:** Collage I and collage III protein expression in lung tissues detected by western blot analysis.

	**1**	**2**	**3**	**4**	**5**	**6**	**7**	**8**	**9**	**10**	**11**	**12**	**13**
COL I	881.3702	649.9987	1832.703	2568.678	2818.209	3147.572	3451.245	3181.307	3539.749	3513.014	3847.802	5188.892	142.3254
COL III	505.3061	484.2094	749.4388	908.0861	1260.177	1496.222	1701.527	1636.657	1462.328	1255.216	1323.994	1795.405	90.23677
GAPDH	4804.041	5780.667	5607.124	5567.676	5250.71	5264.191	5177.216	5562.457	5222.558	4894.892	4387.933	4556.368	4180.9

### Identification of DEGs in PQ -induced human embryonic pulmonary fibrosis

**Figure 4:**
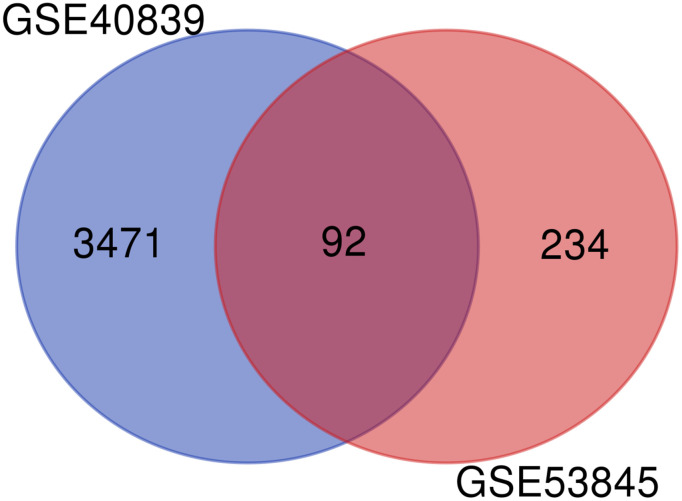
Venn diagram of DEGs in the two GEO datasets.

The GSE40839 and GSE53845 microarray datasets were standardized. The degree of overlap between ontology terms associated with the DEGs in GSE40839 and GSE53845 was high (Figure 4). Moreover, the functional enrichment of these gene sets was analyzed jointly, and 92 overlapping genes between the GSE40839 and GSE53845 datasets were identified (Figure 4).

### Functional enrichment analysis of DEGs

The Gene Ontology (GO) analysis showed that the DEGs were significantly enriched in components involved in ‘nucleic acid binding’,‘CXCR chemokine receptor binding’, ‘cell surface’, and ‘G-protein coupled receptor binding’ (Table 2). To evaluate the biologically functional characteristic of fibrosis-related proteins interaction network, the signaling pathways participated in this biological process were assessed using KEGG pathway enrichment analysis. The biological processes and KEGG pathway analyses demonstrated that the DEGs were enriched in proteins intervening in ‘TNF signaling pathway’, ‘Chemokine signaling pathway’, ‘Jak-STAT signaling pathway’, and ‘p53 signaling pathway’ (Table 2). It’s worth to note that Pathways in fibrosis consisted of TNF signaling pathway (bta04668), Jak-STAT signaling pathway (bta04630) and other signaling pathway involved in lung fibrosis on KEGG website. Additionally, TNF signaling pathway (bta04668) was an essential regulator which acted multi- functional role in cell viability. It linked the main members with various upstream or downstream molecules and other pathways, for example, Chemokine signaling pathway (bta04062), Jak-STAT signaling pathway (bta04630) and p53 signaling pathway (bta04115).

**Table 2:** Functional analysis of the hub genes identified from the protein–protein interaction network.

**Term**	**Description**	**Count**	**P-Value**	**Gene ID**
GO:0003676	nucleic acid binding	26	0.0093558566	FASN,CKM,MTCR,ACACB,PLCL2,PLCE1,PLCB4, ALDH1A1,CKM,AKT3,PLPL3,PLB1,GPLD1,MCTS1, CCL20,MAFKG,LIF,SIK1,RGS1,GADD45B,NFIL3, WWTRI,CXCL2,DUSP1,CEBP,FosL2
GO:0003700	transcription factor activity, sequence-specific DNA binding	14	0.0095883844	ALDH1A1,NFIL3,DUSP1,CKM,AKT3,GADD45B, RGS1,PLB1,JunB,WWTRI,SOCS3,FLT4,PTHIR,CLTR4,
GO:0008083	growth factor activity	5	0.03424423	MTCR,BCL6,IGFBP3,FosL2,IGFBP4
GO:0045236	CXCR chemokine receptor binding	3	0.020546538	RERG,SERPINE2,ATF3
GO:0001664	G-protein coupled receptor binding	5	0.03424423	HBEGF,BCL6,WNT5A,NFIL3,CCL2
GO:0001047	core promoter binding	4	0.027395384	RET,CCL2,ILK,RPTOR
GO:0000987	core promoter proximal region sequence-specific DNA binding	5	0.03424423	CHRNB2,CEBP,FMNL3,ZSWIM5,ATF3
bta04668	TNF signaling pathway	10	0.027395384	JunB,CEBP,C/EBP,DUSP1,ATF3,FosL2,Cidec, CCL2,SOCS3,Fabp4
bta04062	Chemokine signaling pathway	5	0.03424423	TGFB3,TEK,PARD6B,MACF1,KDR
bta04630	Jak-STAT signaling pathway	4	0.027395384	FBLN5,Fabp4,RPTOR,IGFBP4
bta04115	p53 signaling pathway	3	0.020546538	TP53,PDPN,ATF3

GO, Gene Ontology.

### Module analysis from the PPI network

**Figure 5:**
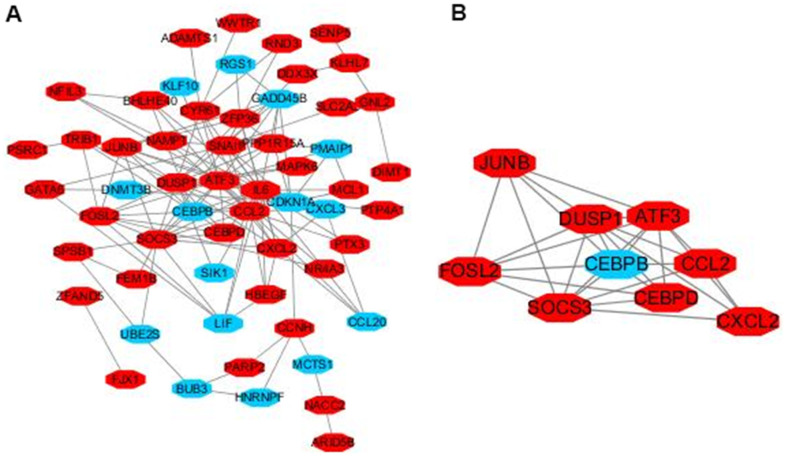
**Module analysis of the PPI network.** Up-regulated genes are marked in light red, down-regulated genes are marked in light blue (**A**). The most significant module generated from the PPI network. JUNB, FOSL2, SOCS3, DUSP1, CCL2, CEBPD, CXCL2, ATF3 protein–protein interaction (**B**). Up-regulated genes are marked in light red, down-regulated genes are marked in light blue.

The interactions of 92 DEGs were identified using the STRING online database. A PPI network was generated with Cytoscape, and the most significant modules were obtained using MCODE (Figure 5A). This analysis revealed AP-1 transcription factor subunit (JunB), fos-like antigen 2 (FosL2), suppressor of cytokine signaling 3 (SOCS3), dual specificity phosphatase 1 (DUSP1), C-C motif chemokine ligand 2 (CCL2), CCAAT/enhancer binding protein (CEBP), chemokine ligand 2 (CXCL2), activating transcription factor 3 (ATF3), and CCAAT/enhancer binding protein (C/EBP) as hub genes (Figure 5B). These genes were closely related to the term ‘transcription factor activity’ and were enriched in ‘TNF signaling pathway’ (Table 2).

## DISCUSSION

Because PQ causes acute lung injuries and subsequent development of pulmonary fibrosis, it ultimately leads to respiratory failure and death [13]. The main known pathological changes associated with PQ poisoning, entering the lung by intranasal or oropharyngeal route, consist of oxidative damages in alveolar cells. This toxic effect triggers lung pathophysiology characterized by extensive deposition of collagen onto the extracellular matrix, release of inflammatory cytokines, and fibroblast proliferation [14, 15]. However, until today, the exact mechanism of PQ-induced lung fibrosis remains largely unclear and no specific treatment drugs for this disease have been approved. Thus, innovative treatment strategies are required to prevent, treat, and even reverse pulmonary fibrosis caused by PQ poisoning.

Importantly, this is the first study to report the development of a preclinical *in vitro* model of PQ-induced pulmonary fibrosis and explore the potential mechanism of development of this disease through bioinformatics analysis. Pulmonary fibrosis is characterized by fibroblast proliferation and abnormal amount of extracellular matrix (ECM) molecules [16, 17]. Lung fibrosis is induced by fibroblasts and myofibroblasts that secrete higher amount of ECM, constituted primarily of collagen types I and III [18, 19]. Therefore, the collagen content in lung tissues can directly reflect the degree of fibrosis. The extent of collagen deposition is reflected by the amount of hydroxyproline content [20], and collagen deposition in local tissues can reflect the severity of the pathology and can be evaluated by collagen staining. Thus, in our study, we detected that in cells exposed to 200 μmol/L PQ for 48 hours, the expression of collagen I and α-SMA proteins was significantly upregulated. In other words, on exposure to PQ at a concentration of 200 μmol/L, MRC-5 developed signs of severe pulmonary fibrosis. Thus, we found that PQ exposure significantly upregulated collage I, collagen III, and SAM expression in lung cells. These data indicate that the injuries caused by PQ in the lung tissues activate TNF signaling by recruiting inflammatory factors into the lung cells, in line with results from a previous study [21]. In previous experiments and in this preliminary study, we initially found that under the aforementioned experimental conditions, the proportion of cell necrosis was low. The expression of fibrosis-related proteins needs further mechanistic studies and intervention experiments.

Previous studies have demonstrated that TNF-α causes significant damages to lung tissues [22, 23] and is an important regulator of cell proliferation [24–26]. Christopher et al. [27] found that TNF-R2 can also independently activate JNK (c-jun N-terminal protein kinase) and ERK (extracellular signal 2 regulated protein kinase) in lung tissues, indicating that TNF-R2 not only has "ligand transmission" function, but also transmits signals independently. PQ can activate inflammatory cells such as macrophages and neutrophils to secrete a large spectrum of inflammatory factors, and thereby, to participate in the occurrence of pulmonary fibrosis. Previous studies have suggested that the levels of tumor necrosis factor α (TNF-α), nuclear factor (NF-κB), interleukin 1β (IL-1β), and IL-6 increase in the bronchoalveolar lavage fluid of experimental rats with acute PQ-induced lung injury [28]. Nine hub genes (JUNB, FOSL2, SOCS3, DUSP1, CEBPB, CEBPD, ATF3, CCL2, and CXCL2) were identified as having the highest scores in the PPI network. JUNB, which is strongly dependent on the AP1 factor, could regulate collagen type 1 and collagen type II in pulmonary fibrosis [29]. DUSP1 was associated with increased expression of collagen I, collagen IV, and fibronectin [30]. CEBPB and CEBPD could act on bleomycin-induced fibrosis [31]. ATF3, a member of the integrated stress response (ISR), negatively regulates PINK1 (PTEN Induced Kinase 1) transcription. ATF3 in type II lung epithelial cells accelerates PQ-induced lung fibrosis in mouse [32]. CCL2, which is produced by AECs, promotes fibrosis through CCR2 activation. CCR2 signaling is critical for the initiation and progression of pulmonary fibrosis partly through the recruitment of pro-fibrotic bone marrow-derived monocytes [33, 34]. Chemokine receptor type 2 (CXCR2), which is a chemokine highly expressed in the lung, exhibits inflammatory and fibrotic effects [35]. Therefore, TNF modulators can potentially act as therapeutic targets in PQ-induced pulmonary fibrosis. This study revealed that the expression levels of JUNB, FOSL2, SOCS3, DUSP1, CEBPB, CEBPD, ATF3, CCL2, and CXCL2 were significantly higher in lung fibrosis and could promote the disease [36]. Thus, our findings may provide new insights into potential gene-targeting therapeutic approaches to regulate PQ-induced pulmonary toxicity.

Nevertheless, there are some defects in this study. The experimental results were obtained in a single experimental setup and will need to be strengthened in other systems. Moreover, our experimental groups were relatively small. Finally, this study describes phenomena associated with PQ-induced fibrosis using bioinformatics analyses but needs to be completed by specific mechanistic experiments. Further research is needed to confirm and explore the mechanisms underlying PQ-induced lung fibrosis.

## CONCLUSIONS

In summary, for the first time, our study successfully established a preclinical *in vitro* model of PQ-induced pulmonary fibrosis based on MRC-5 cells, and defined the highest non-lethal concentration of PQ at 200 μmol/L until 48-hour exposure. Furthermore, we uncovered TNF signaling pathway and nine hub genes as potentially involved in PQ-induced lung fibrosis progression. Our study may aid the design of novel approaches for the control and treatment of PQ-induced pulmonary fibrosis.
